# Prognostic value of systemic inflammatory response index in patients with acute coronary syndrome undergoing percutaneous coronary intervention

**DOI:** 10.1080/07853890.2022.2083671

**Published:** 2022-06-13

**Authors:** Kangning Han, Dongmei Shi, Lixia Yang, Zhijian Wang, Yueping Li, Fei Gao, Yuyang Liu, Xiaoteng Ma, Yujie Zhou

**Affiliations:** a Beijing Anzhen Hospital, Capital Medical University, Beijing, China; b Beijing Institute of Heart, Lung and Blood Vessel Disease, Beijing, China

**Keywords:** Systemic inflammatory response index, acute coronary syndrome, percutaneous coronary intervention, cardiovascular outcomes

## Abstract

**Background:**

The systemic inflammatory response index (SIRI) is a novel inflammatory biomarker in many diseases.

**Objectives:**

The aim of this study was to examine the association between SIRI and adverse events in patients with the acute coronary syndrome (ACS) undergoing percutaneous coronary intervention.

**Methods:**

A total of 1724 patients with ACS enrolled from June 2016 to November 2017 at a single centre were included in this study, and SIRI was calculated for each patient. The primary endpoint was the composite of major adverse cardiovascular events (MACE), including overall death, non-fatal myocardial infarction, non-fatal stroke, and unplanned repeat revascularization.

**Results:**

During a median follow-up of 927 days, 355 patients had MACE. Multivariate Cox analysis showed that SIRI was significantly associated with MACE (hazard ratio: 1.127, 95% confidence interval: 1.034–1.229 *p* = .007). The results were consistent in multiple sensitivity analyses. The addition of SIRI had an incremental effect on the predictive ability of the Global Registry of Acute Coronary Events risk score for MACE (integrated discrimination improvement: 0.007, *p* = .040; net reclassification improvement: 0.175, *p* = .020; likelihood ratio test: *p* < .001). The restricted cubic spline showed a monotonic increase with a greater SIRI value for MACE (*p* < .001).

**Conclusion:**

SIRI was an independent risk factor for MACE and provided incremental prognostic information in patients with ACS undergoing percutaneous coronary intervention.

**KEY MESSAGES**
- The SIRI is a strong and independent risk factor for adverse outcomes in patients with ACS undergoing percutaneous coronary intervention.
- Higher SIRI is associated with a more severe disease status.
- The SIRI could increase the prognostic value of the GRACE risk score.

## Introduction

With a global prevalence of 154 million in 2016, coronary artery disease (CAD) represents 32.7% of the global burden of cardiovascular disease and is one of the leading causes of death [1]. Atherosclerosis, the main cause of CAD, is an inflammatory process wherein the immune response interacts with metabolic disorders to generate and activate coronary lesions. Much evidence has suggested that chronic inflammation plays a critical role in the pathogenesis of atherosclerosis [2,3]. Immune cells, including neutrophils, monocytes, lymphocytes, and mast cells, infiltrate the atherosclerotic lesions and initiate a cytokine cascade [4]. A larger number of white blood cells is associated with the onset and poor prognosis of CAD [5,6]. Inflammation also incites plaque destabilization and precipitates acute coronary syndrome (ACS) [7].

As a reflection of inflammation, peripheral blood inflammatory cell count and its derived indicators are now widely used in clinical practice. These indicators are considered to be inexpensive and easily accessible biomarkers that are associated with increased risk of CAD [8,9], stroke [10], and overall death [11]. For example, studies have found that neutrophil to lymphocyte ratio (NLR), monocyte to lymphocyte ratio (MLR), and monocyte to high-density lipoprotein cholesterol (HDL-C) ratio have strong predictive roles [12–14]. Recently, a novel indicator has emerged called the systemic inflammatory response index (SIRI). SIRI is a composite index based on the absolute count of three different inflammatory cells, namely, neutrophils, monocytes, and lymphocytes, and it is highly associated with cancer, hyperuricaemia, rheumatoid arthritis, and stroke [15–18]. Elevated SIRI values are related to an increased risk of myocardial infarction (MI) and overall death [19]. However, whether SIRI is an independent risk factor for adverse prognosis in patients with ACS is still unknown. Here, we investigated the prognostic value of SIRI in ACS patients undergoing percutaneous coronary intervention (PCI).

## Method

This study was a single-centre, retrospective analysis derived from a prospective observational study (ChiCTR1800017417) of patients with ACS undergoing PCI conducted between June 2016 and November 2017 at Beijing Anzhen Hospital. A total of 1770 patients were consecutively enrolled and followed up after 1 month and every 6 months thereafter. The exclusion criteria of this study included patients with infection or rheumatic disease or a history of coronary artery bypass graft surgery. Eventually, 1724 patients were included in the final analysis, with a median follow-up of 927 days. This study was performed following the Helsinki Declaration of Human Rights and was approved by the institutional review board of Beijing Anzhen Hospital, Capital Medical University. Written informed consent was obtained from all of the patients.

Data on demographics, medical history, angiographic information, and medicine intake were collected from the medical records system using a questionnaire. The SIRI was defined as neutrophils × monocytes/lymphocytes. Smoking status was defined as currently smoking or not smoking. ACS was classified into unstable angina, ST-segment elevation myocardial infarction (STEMI), and non-STEMI (NSTEMI). MI was defined as an elevated level of creatine kinase or cardiac troponin higher than the upper limit of the normal range accompanied by either ischaemic symptoms or electrocardiographic changes implicating ischaemia.

Hypertension was diagnosed as systolic blood pressure ≥140 mmHg and/or diastolic blood pressure (DBP) ≥90 mmHg and/or receiving anti-hypertension treatment. Fasting plasma glucose (FPG) ≥7.0 mmol/L and/or casual plasma glucose ≥11.1 mmol/L, and/or 2-h blood glucose after an oral glucose tolerance test ≥11.1 mmol/L, and/or using antidiabetic medicine were considered to indicate diabetes. Dyslipidemia was defined as a fasting serum total cholesterol >5.17 mmol/L, low-density lipoprotein cholesterol (LDL-C) >3.36 mmol/L, triglyceride >1.69 mmol/L, HDL-C <1.03 mmol/L, and/or use of lipid-lowering drugs. Hyperuricaemia was defined when uric acid was >416 μmol/L in men and >357 μmol/L in women. Patients with previous ischaemic strokes or transient ischaemic attacks were defined as having a history of cerebrovascular disease. Chronic kidney disease (CKD) was defined as the estimated glomerular filtration rate (eGFR) <60 mL/min/1.73 m^2^, calculated using the Chronic Kidney Disease Epidemiology Collaboration equation. Patients with congestive heart failure signs/symptoms, ongoing heart failure therapy, or left ventricular ejection fraction (LVEF) <40% were defined as having heart failure. The Synergy between PCI with TAXUS and Cardiac Surgery (SYNTAX) score and the Global Registry of Acute Coronary Events (GRACE) risk score were calculated for each patient.

Information on adverse events was obtained through telephone contact or the medical records system. The primary endpoint was the composite of major adverse cardiovascular events (MACE) including overall death, non-fatal MI, non-fatal stroke, and unplanned repeat revascularization (URR). Non-fatal MI was defined as an elevated level of cardiac creatine kinase or cardiac troponin accompanied by either ischaemic symptoms or electrocardiographic changes. Non-fatal MI was also diagnosed when patients had new pathological Q waves in ≥2 contiguous electrocardiogram leads. Patients with an acute ischaemic cerebral vascular event underlying a neurological event were diagnosed with stroke. Only Q-wave MI was considered MI within 1 week after the index PCI. Any non-staged revascularization after the index PCI was considered URR. If the same event occurred more than twice, the first event was used in the analysis. The most severe endpoint event was selected for the analysis if >1 event occurred during follow-up (death > stroke > MI > URR).

Continuous variables are summarised as mean ± standard deviation or median (interquartile range). Comparisons were performed using the *t*-test or Mann–Whitney *U*-test according to the distribution. Categorical variables are expressed as the number (percentage) for which the chi-square test or Fisher’s exact test was used accordingly. Receiver operating characteristic (ROC) curve analysis was performed to determine the cut-off value of SIRI for MACE, and the area under the ROC curve was also calculated. SIRI was analyzed as continuous and categorical (tertiles) variables. Cumulative incidence curves were visualized using the Kaplan–Meier analyses, and the log-rank test was performed. Cox proportional hazard analyses with six models were performed to detect independent risk factors by computing the hazard ratio with a 95% confidence interval. Subgroup analyses according to age, sex, hypertension, diabetes, dyslipidemia, and ACS type were carried out. Restricted cubic spline (RCS) with 4 knots based on model 6 in the multivariate analysis was used to evaluate the association between the SIRI and MACE. To analyze the incremental value of SIRI, we introduced the SIRI into the GRACE risk score and compared their performance, for which the C-statistic, net reclassification improvement (NRI) index, and integrated discrimination improvement (IDI) index were used to evaluate the discrimination properties. The likelihood ratio (LR) test, Akaike information criterion (AIC), and Bayesian information criterion (BIC) were calculated to assess calibration properties. The statistical analyses were performed using IBM SPSS version 26.0 (IBM Corp., Armonk, NY, USA), R version 3.6.1 (The R Project for Statistical Computing, Vienna, Austria), and MedCalc version 20.0.1 (MedCalc Software, Ostend, Belgium).

## Results

Among the 1724 patients with a median follow-up of 927 days, the mean age was 60 ± 10 years, and 1323 (76.74%) patients were men. The baseline characteristics of the participants are summarised in Table 1. Patients with higher tertiles of SIRI were older and more likely to be male, and these patients had higher percentages of smoking, dyslipidemia, previous MI, heart failure, and the presence of STEMI. These patients also had higher neutrophil and monocyte counts, high-sensitivity C-reactive protein (hs-CRP), FPG, and SYNTAX scores but lower DBP, LVEF, lymphocyte count, total cholesterol, HDL-C, and eGFR. In angiography, patients with higher SIRI tertiles had a higher SYNTAX score and more frequent left anterior descending coronary artery intervention and a lower percentage of complete revascularization. As for medicine intake, these patients had a lower percentage of aspirin intake and higher percentages of angiotensin-converting enzyme inhibitor/angiotensin receptor blocker and β-blocker prescriptions at discharge.

**Table 1.** Baseline characteristics of the study population according to the SIRI tertiles.

Variables	T1: <0.63	T2: 0.63–1.02	T3: >1.02	*p* Value
SIRI	0.46 ± 0.12	0.81 ± 0.11	1.74 ± 1.06	<.001
Age (years)	59 ± 10	60 ± 11	61 ± 11	.019
Male sex, *n* (%)	380 (66.2)	458 (79.5)	485 (84.5)	<.001
BMI (kg/m^2^)	25.66 ± 2.99	25.84 ± 3.03	25.56 ± 3.22	.297
SBP (mmHg)	130 ± 15	130 ± 16	130 ± 18	.926
DBP (mmHg)	77 ± 9	76 ± 11	75 ± 12	.022
Risk factors				
Smoking, *n* (%)	218 (38.0)	271 (47.0)	272 (47.4)	.001
Hypertension, *n* (%)	353 (61.5)	370 (64.2)	376 (65.6)	.353
Diabetes, *n* (%)	269 (46.9)	263 (45.7)	260 (45.3)	.856
Dyslipidemia, *n* (%)	439 (76.5)	470 (81.6)	469 (81.7)	.041
Hyperuricaemia, *n* (%)	108 (18.8)	133 (23.1)	130 (22.6)	.153
Cancer, *n* (%)	2 (0.3)	6 (1.0)	5 (0.9)	.367
Previous MI, *n* (%)	88 (15.3)	119 (20.7)	123 (21.4)	.017
Previous PCI, *n* (%)	104 (18.1)	120 (20.8)	117 (20.4)	.465
Previous CVD, *n* (%)	28 (4.9)	29 (5.0)	43 (7.5)	.105
CKD, *n* (%)	13 (2.3)	15 (2.6)	25 (4.4)	.088
Heart failure, *n* (%)	31(5.4)	32 (5.6)	59 (10.3)	.001
LVEF (%)	65 (61–68)	65 (60–68)	63 (58–68)	<.001
Clinical presentation				
UA, *n* (%)	484 (84.3)	417 (72.4)	378 (65.9)	<.001
NSTEMI, *n* (%)	57 (9.9)	86 (14.9)	77 (13.4)	.034
STEMI, *n* (%)	33 (5.7)	73 (12.7)	119 (20.7)	<.001
GRACE risk score	79 (65–91)	82 (66–100)	87 (73–107)	<.001
Laboratory results				
Neutrophil (×10^6^/μL)	3.07 (2.61–3.61)	3.94 (3.42–4.55)	5.18 (4.40–6.06)	<.001
Monocyte count (×10^6^/μL)	0.29 (0.22–0.33)	0.35 (0.30–0.42)	0.48 (0.40–0.58)	<.001
Lymphocyte (×10^6^/μL)	1.89 (1.58–2.34)	1.73 (1.42–2.14)	1.57 (1.31–2.00)	<.001
hs-CRP (mg/L)	0.93 (0.45–2.05)	1.32 (0.67–2.86)	2.43 (0.92–6.68)	<.001
Total cholesterol (mmol/L)	4.23 ± 1.00	4.11 ± 0.96	4.10 ± 1.00	.045
LDL-C (mmol/L)	2.48 ± 0.83	2.41 ± 0.79	2.43 ± 0.80	.274
HDL-C (mmol/L)	1.08 ± 0.24	1.01 ± 0.22	1.00 ± 0.24	<.001
Triglycerides (mmol/L)	1.43 (0.99–1.99)	1.49 (1.04–2.10)	1.45 (1.01–2.12)	<.001
FPG (mmol/L)	6.22 ± 1.64	6.32 ± 1.72	6.49 ± 1.79	.026
Glycosylated haemoglobi*n* (%)	6.10 (5.60–7.03)	6.05 (5.60–7.10)	6.10 (5.60–7.20)	.300
eGFR (mL/min/1.73 m^2^)	93.19 ± 14.04	93.87 ± 13.50	90.55 ± 15.70	<.001
Angiographic findings				
Left-main and/or multivessel disease, *n* (%)	477 (83.1)	486 (84.4)	498 (86.8)	.216
Chronic total occlusion, *n* (%)	123 (21.4)	122 (21.2)	120 (20.9)	.977
Lesions with length >20 mm, *n* (%)	299 (52.1)	284 (49.3)	322 (56.1)	.068
Bifurcation or trifurcation lesions, *n* (%)	428 (74.6)	440 (76.4)	431 (75.1)	.761
SYNTAX score	18 (12–26)	20 (12–28)	22 (14–32)	<.001
Procedural results				
Target vessel-LM, *n* (%)	17 (6.3)	21 (8.0)	18 (6.9)	.751
Target vessel-LAD, *n* (%)	117 (43.5)	143 (54.4)	137 (52.7)	.026
Target vessel-LCX, *n* (%)	71 (26.4)	75 (28.5)	78 (30.0)	.651
Target vessel-RCA, *n* (%)	119 (44.2)	102 (38.8)	102 (39.2)	.363
Complete revascularization, *n* (%)	380 (66.2)	358 (62.2)	320 (55.7)	.001
Periprocedural medications				
Low molecular weight heparin, *n* (%)	368 (64.1)	380 (66.0)	389 (67.8)	.425
Bivalirudin, *n* (%)	69 (12.0)	64 (11.1)	63 (11.0)	.832
GP IIb/IIIa receptor antagonist, *n* (%) (17.0) 42 (22.3)	111 (19.3)	129 (22.4)	129 (22.5)	.336
Prescription at discharge				
Aspirin, *n* (%)	573 (99.8)	575 (99.8)	560 (97.6)	<.001
Clopidogrel, *n* (%)	519 (90.4)	534 (92.7)	530 (92.3)	.315
Ticagrelor, *n* (%)	55 (9.6)	42 (7.3)	44 (7.7)	.321
Statins, *n* (%)	574 (100.0)	576 (100.0)	574 (100.0)	.135
ACEI/ARBs, *n* (%)	256 (44.6)	254 (44.1)	320 (55.7)	<.001
β-blockers, *n* (%)	384 (66.9)	403 (70.0)	426 (74.2)	.024
Oral anticoagulants, *n* (%)	4 (0.7)	4 (0.7)	0 (0.0)	.135
Nitroglycerin, *n* (%)	412 (71.8)	436 (75.7)	444 (77.4)	.082
Calcium channel blockers, *n* (%)	131 (22.8)	156 (27.1)	145 (25.3)	.247

SIRI: systemic inflammatory response index; BMI: body mass index; SBP: systolic blood pressure; DBP: diastolic blood pressure; MI: myocardial infarction; PCI: percutaneous coronary intervention; CVD: cerebrovascular disease; CKD: chronic kidney disease; LVEF: left ventricular ejection fraction; UA: unstable angina; NSTEMI: non ST-segment elevation myocardial infarction; STEMI: ST-segment elevation myocardial infarction; GRACE: Global registry of acute coronary events; hs-CRP: high sensitive C-reactive protein; LDL-C: low-density lipoprotein-cholesterol; HDL-C: high-density lipoprotein-cholesterol; FPG: fasting plasma glucose; eGFR: estimated glomerular filtration rate; SYNTAX: Synergy between PCI with TAXUS and Cardiac Surgery; LM: left main artery; LAD: left anterior descending artery; LCX: left circumflex artery; RCA: right coronary artery; ACEI: angiotensin converting enzyme inhibitor; ARB: angiotensin receptor blocker.

**Figure 1.**
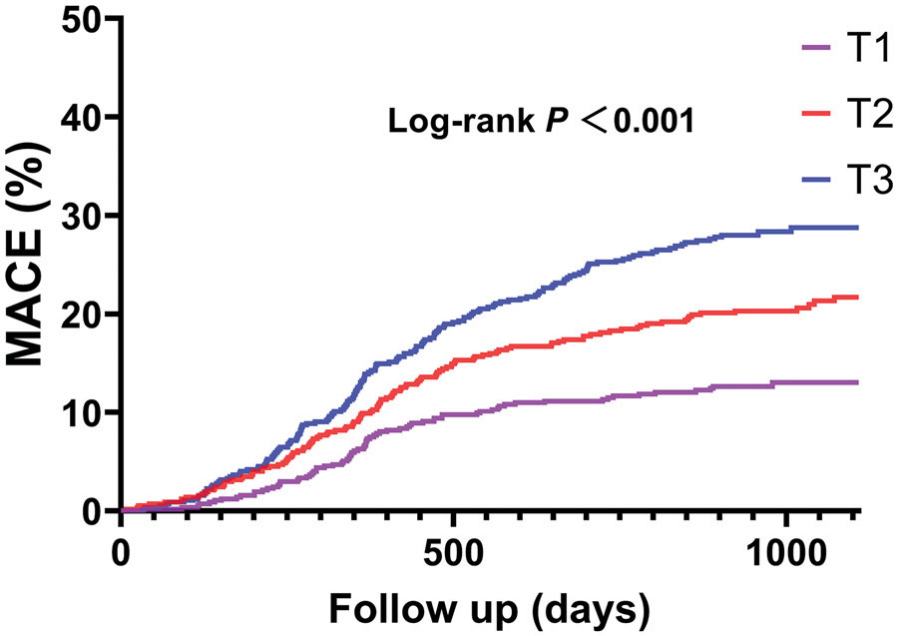
Cumulative incidence plot of the MACE stratified by SIRI tertiles. MACE included overall death, non-fatal MI, non-fatal stroke, and URR. MI: myocardial infarction; URR: unplanned repeat revascularization.

**Figure 2.**
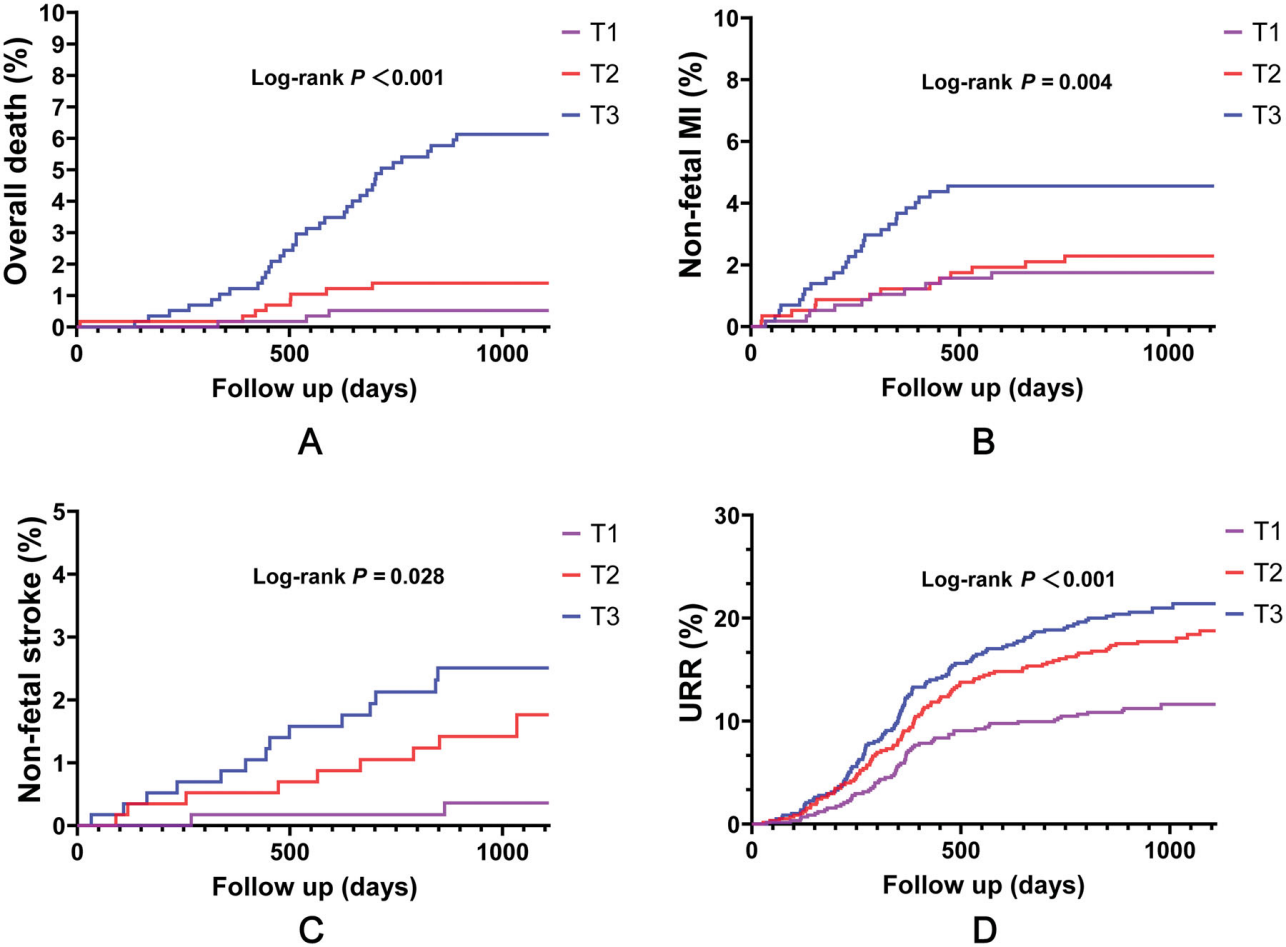
Cumulative incidence plots of each component of MACE stratified by SIRI tertiles. (A) overall death; (B) non-fatal stroke; (C) non-fatal MI; (D) URR. MI: myocardial infarction; URR: unplanned repeat revascularization.

At follow-up, patients with higher SIRI tertiles had a higher percentage of MACE (Table 2). These patients had higher percentages of overall death, non-fatal MI, non-fatal stroke, and URR (*p* < .05 for all). Kaplan–Meier survival analysis showed that the cumulative incidence of MACE increased with higher SIRI tertiles (log-rank test, *p* < .001) (Figure 1). Considering each component of MACE, patients with higher SIRI tertiles had a higher cumulative incidence of overall death (*p* < .001), non-fatal MI (*p* = .004), non-fatal stroke (*p* = .028), and URR (*p* < .001) (Figure 2). When considering SIRI as a continuous variable, SIRI was significantly associated with MACE in both univariate and multivariate Cox regression analyses (*p* < .05 for all models) (Table 3). Additionally, when considering SIRI as a categorical variable, this association was also significant (*p* < .05 for all models) (Table 3).

**Table 2.** Adverse cardiovascular events according to SIRI tertiles during follow-up.

Adverse cardiovascular events	T1: <0.63	T2: 0.63–1.02	T3: >1.02	*p* Value
MACE, *n* (%)	73 (12.7)	120 (20.8)	162 (28.2)	<.001
Overall death, *n* (%)	3 (0.5)	8 (1.4)	35 (6.1)	<.001
Non-fatal MI, *n* (%)	10 (1.7)	13 (2.3)	26 (4.5)	.010
Non-fatal stroke, *n* (%)	2 (0.3)	9 (1.6)	14 (2.4)	.012
URR, *n* (%)	65 (11.3)	104 (18.1)	118 (20.6)	<.001

The MACE was defined as the composite of overall death, non-fatal stroke, non-fatal MI, and URR.

MACE: major adverse cardiovascular events; MI: myocardial infarction; URR: unplanned repeat revascularization.

**Table 3.** Univariate and multivariate Cox proportional hazard analyses for the MACE according to the SIRI value.

	SIRI as a continuous variable				SIRI as a categorical variable		
	HR	95% CI	*p* Value		HR	95 CI%	*p* Value
Model 1	1.218	1.138–1.303	<.001	T1	Reference		
				T2	1.700	1.271–2.274	<.001
				T3	2.402	1.824–3.169	<.001
Model 2	1.213	1.133–1.299	<.001	T1	Reference		
				T2	1.718	1.282–2.303	<.001
				T3	2.437	1.838–3.231	<.001
Model 3	1.203	1.120–1.292	<.001	T1	Reference		
				T2	1.654	1.231–2.223	.001
				T3	2.374	1.782–3.164	<.001
Model 4	1.145	1.054–1.243	.001	T1	Reference		
				T2	1.572	1.168–2.115	.003
				T3	2.132	1.585–2.866	<.001
Model 5	1.114	1.022–1.214	.014	T1	Reference		
				T2	1.565	1.162–2.107	.003
				T3	1.944	1.443–2.618	<.001
Model 6	1.127	1.034–1.229	.007	T1	Reference		
				T2	1.582	1.174–2.132	.003
				T3	1.999	1.482–2.696	<.001

Model 1: Unadjusted.

Model 2: Adjusted for age, sex.

Model 3: Model 2 + smoking, hypertension, diabetes, dyslipidemia, cancer, hyperuricaemia, previous MI, previous PCI, previous CVA, type of ACS.

Model 4: Model 3 + hs-CRP, HDL-C, LDL-C, total cholesterol, triglycerides.

Model 5: Model 4 + GRACE risk score, SYNTAX score, complete revascularization.

Model 6: Model 5 + aspirin at discharge + ACEI/ARBs at discharge + β blocker at discharge.

**Figure 3.**
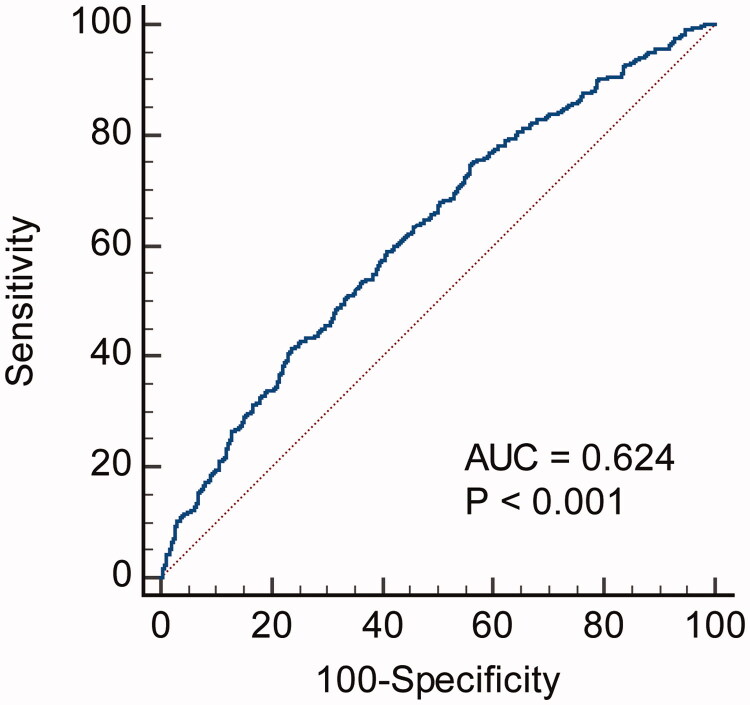
ROC analysis shows the predictive value of SIRI for MACE.

In view of each specific adverse event, SIRI as a continuous variable was associated with overall death, non-fatal MI, and URR in univariate Cox regression analyses (Table 4). In multivariate analyses, SIRI exhibited an association with non-fatal MI and URR. ROC curve analysis showed that the C-index of SIRI in predicting MACE was 0.624, with a sensitivity of 75.2% and specificity of 43.6% (Figure 3). Moreover, compared with each specific component of SIRI (neutrophils, monocytes, and lymphocytes), the SIRI has the highest C-index (Supplemental Table 1). As a categorical variable according to tertiles, SIRI was associated with overall death, non-fatal MI, non-fatal stroke, and URR in univariate analyses. In multivariate analyses, the SIRI was associated with overall death, non-fatal MI, and URR.

**Table 4.** Univariate and multivariate Cox proportional hazard analyses for adverse prognosis according to the SIRI.

		Univariate analyses			Multivariate analyses		
		HR	95%CI	*p* Value	HR	95%CI	*p* Value
SIRI – categorical variable							
Overall death	T1	Reference			Reference		
	T2	2.662	0.706–10.034	.148	2.272	0.590–8.752	.233
	T3	11.912	3.664–38.730	<.001	7.206	2.080–24.963	.002
Non-fatal MI	T1	Reference			Reference		
	T2	1.300	0.570–2.964	.533	1.339	0.580–3.092	.494
	T3	2.665	1.285–5.525	.008	2.384	1.078–5.271	.032
Non-fatal stroke	T1	Reference			Reference		
	T2	4.480	0.968–20.737	.055	4.218	0.892–19.944	.069
	T3	7.177	1.631–31.580	.009	4.659	0.960–22.610	.056
URR	T1	Reference			Reference		
	T2	1.643	1.205–2.240	.002	1.554	1.130–2.138	.007
	T3	1.940	1.433–2.627	<.001	1.724	1.245–2.388	.001
SIRI – continuous variable							
Overall death		1.364	1.207–1.541	<.001	1.096	0.908–1.324	.339
Non-fatal MI		1.325	1.158–1.515	<.001	1.235	1.030–1.480	.022
Non-fatal stroke		1.196	0.905–1.580	.208	0.961	0.555–1.663	.887
URR		1.175	1.078–1.282	<.001	1.129	1.015–1.255	.025

Multivariate analyses were based on model 6 in Table 3.

MACE: major adverse cardiovascular events; MI: myocardial infarction; URR: unplanned repeat revascularization.

**Figure 4.**
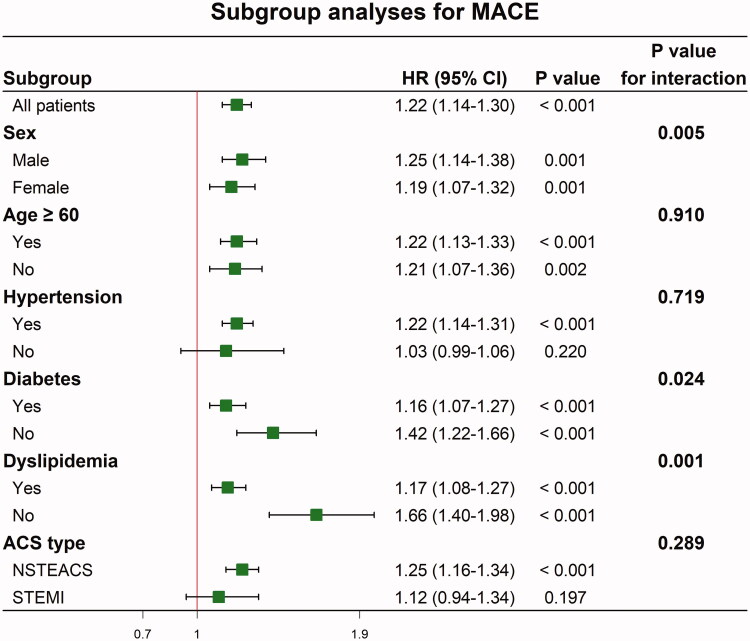
Subgroup analyses of continuous SIRI for MACE.

**Figure 5.**
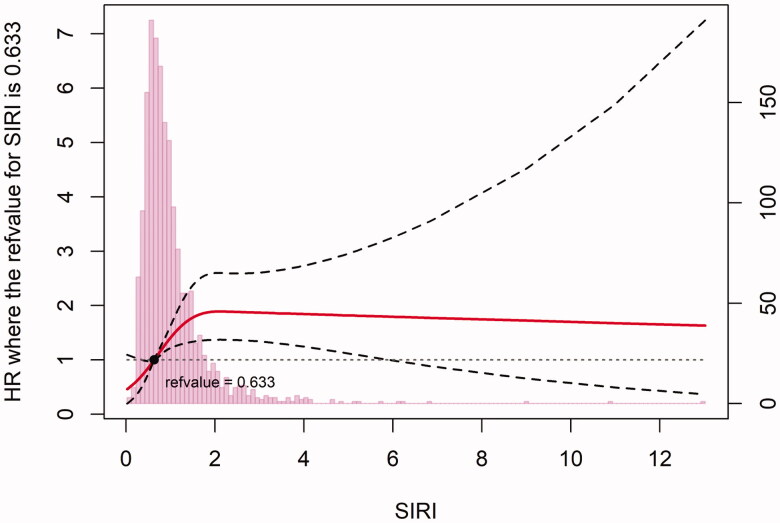
Restricted cubic spline demonstrated the association between SIRI and MACE.

Table 5 shows that the inclusion of SIRI improved MACE prediction compared with the GRACE risk score. SIRI increased the C-statistic value and showed significant improvement in the NRI and IDI. SIRI also increased the calibration properties as indicated in the LR test (*p* < .001), AIC, and BIC. Subgroup analyses according to sex, age, hypertension, diabetes, dyslipidemia, and ACS type are shown in Figure 4. In patients presenting with STEMI or without hypertension, SIRI was not significantly associated with MACE. The restricted cubic spline with 4 knots based on multivariate analysis of model 6 showed that SIRI was positively associated with MACE (*p* < .001) (Figure 5).

**Table 5.** Model performance after the addition of SIRI to the GRACE risk score for predicting clinical outcomes.

	MACE		Death		Death + MI	
	GRACE	GRACE + SIRI	GRACE	GRACE + SIRI	GRACE	GRACE + SIRI
Discrimination						
C-statistic	0.527	0.601	0.681	0.742	0.613	0.688
*p*-Value	<.001		<.001		<.001	
IDI (95% CI)	0.007 (0.001 to 0.020)		−0.001 (−0.002 to 0.004)		0.189 (0.074 to 0.292)	
*p*-Value	.040		.851		<.001	
NRI (95% CI)	0.175 (0.027 to 0.215)		−0.001 (−0.002 to 0.004)		−0.001 (−0.001 to 0.012)	
*p*-Value	.020		.059		.119	
Calibration						
LR test, *p*-value	<.001		.018		<.001	
AIC	5195	5179	661	658	1307	1296
BIC	5199	5188	663	661	1309	1301

MACE: major adverse cardiovascular events; GRACE: Global registry of acute coronary events; MI: myocardial infarction; IDI: integrated discrimination improvement; NRI: net reclassification improvement; LR: likelihood ratio; AIC: Akaike information criterion; BIC: Bayesian information criterion.

## Discussion

This study was focussed on the association of SIRI with adverse prognosis in patients with ACS undergoing PCI, and the remarkable significance of SIRI was revealed. Specifically, we found a quantitative association between SIRI and MACE. Moreover, SIRI increased the prognostic role of the GRACE risk score and was independent of hs-CRP.

The inflammatory process in atherosclerosis is dominated by the innate immune response in the early stage of plaque formation. As a precursor of foam cells, monocytes transform into macrophages, proliferate, and phagocytose oxidized LDL-C, ultimately forming the lipid core of plaque [20]. Monocytes also promote destabilization of the fibrous cap, participating in plaque rupture through secreting lytic enzymes, such as matrix metalloproteinases [21]. Monocytes are also involved in thrombus propagation, leading to a coagulation cascade [22]. The number of monocytes is negatively associated with the extent of myocardial salvage and recovery of left ventricular function after MI [23]. Inhibition of monocyte infiltration and differentiation could attenuate early atherogenesis [24].

Secretion by neutrophils of reactive oxygen species and proteases results in the activation and dysregulation of the endothelial layer, resulting in LDL-C extravasation and promoting foam cell formation. Neutrophils localize near plaques, especially those with rupture-prone features. During the atherosclerosis process, neutrophils can also enhance monocyte adhesion and transmigration. Epidemiological studies have shown that circulating neutrophil count positively correlates with coronary atherosclerotic risk [25]. A higher neutrophil count is an independent predictor of adverse cardiovascular events [26]. Infiltration of neutrophils into advanced lesions has been shown to induce collagen degradation and necrosis [27]. Histopathologic studies show that neutrophil count is strongly associated with the formation of rupture-prone lesions, suggesting a role in plaque destabilization [28]. A lower lymphocyte level has been associated with poor cardiovascular outcomes in ACS [29]. Lymphocytopenia is independently associated with the occurrence of mechanical complications after MI [30]. Patients with heart failure who have a lower lymphocyte count have a higher mortality rate [31]. Higher MLR has been associated with the occurrence of heart failure, myocarditis, and coronary severity [32–34]. In stable patients with CAD, lower lymphocyte count is associated with a higher mortality rate [35].

Inflammation plays an important role in the pathogenesis of CAD and especially ACS. At present, many inflammatory indicators such as hs-CRP, neutrophil count, and monocyte count are associated with patient prognosis in CAD. However, a single indicator of inflammation is not sufficient to predict the severity of inflammation. The SIRI, which combines three inflammatory biomarkers, is a comprehensive, easily accessible, and inexpensive indicator of chronic low-grade inflammation. SIRI also includes the NLR and MLR, which might be a more sensitive and useful inflammatory biomarkers than any of these alone. Indeed, SIRI is associated with many diseases. A higher SIRI is positively associated with the risk and poor prognosis of stroke [17,19]. SIRI has also been evaluated as a biomarker in the diagnosis and assessment of rheumatoid arthritis [18]. In patients with cancer, SIRI could predict postoperative survival and disease recurrence [36,37]. In this study, we showed that a higher SIRI was correlated with poor clinical presentation, where patients with a higher SIRI had higher percentages of smoking, dyslipidemia, history of MI, heart failure, and STEMI and a higher SYNTAX score. These patients also had lower LVEF, HDL-C and eGFR. A higher SIRI may be associated with more severe low-grade inflammation. Similarly, NLR is negatively correlated with HDL-C and positively correlated with the SYNTAX score [38,39]. Higher NLR is associated with the presence of diabetes and CKD [40,41]. MLR is also positively correlated with the SYNTAX score and vulnerability of coronary lesions [42,43].

As the combination of NLR and MLR, SIRI may have similar scenarios of treatment. Previous studies investigated treatment for patients with a high NLR. Neither proprotein convertase subtilisin/Kexin type-9 inhibitors nor rosuvastatin or bococizumab could significantly decrease the NLR [12]. Nebivolol, but not metoprolol, has antioxidant and anti-inflammatory properties and could decrease the NLR [44]. Additionally, amlodipine and valsartan could decrease the NLR in patients with newly diagnosed hypertension [45]. In patients with hypercholesterolaemia, atorvastatin could significantly decrease the NLR [46]. Inhibition of interleukin-1β with canakinumab could lower the NLR in a dose-dependent response and reduce cardiovascular events [47,48]. Colchicine, which can inhibit the inflammatory response mediated by monocytes and neutrophils, could reduce MACE in patients with CAD [49,50]. Methotrexate cannot reduce the inflammatory response and has no effects on cardiovascular events [51]. No studies have investigated interventions in terms of a high SIRI; therefore, further studies on lowering the SIRI are needed.

Different kinds of predictive risk scores have been developed in ACS, among which the GRACE and Thrombolysis in Myocardial Infarction (TIMI) risk scores are the most widely used. In particular, studies have shown that the GRACE score is more efficient than the TIMI score [52,53]. However, the GRACE risk score does not include inflammatory indicators. Some potential risk factors beyond the GRACE risk score system have been identified, and these factors improve the predictive ability of adverse events after ACS. Considering the inflammatory biomarkers, hs-CRP and monocyte to HDL-C ratio could improve the predictive power of the GRACE risk score [14,54]. In this study, we showed that the novel biomarker SIRI could also improve the prognostic value of the GRACE risk score in patients with ACS undergoing PCI. Several bleeding risk scores include the Can Rapid risk stratification of Unstable angina patients Suppress Adverse outcomes with Early implementation of the ACC/AHA Guidelines (CRUSADE) and Acute Catheterisation and Urgent Intervention Triage Strategy (ACUITY) scores. However, whether SIRI has predictive value in bleeding and whether it can improve the predictive value of CRUSADE and ACUITY scores are still unknown; further investigation is needed.

## Limitations

First, this was a single centre study based on the Chinese population, and there was no external validation of SIRI in other populations; therefore, the general application of the findings should be made with caution. Second, future studies on long-term events are needed. Third, the predictive value of SIRI based on the variates measured at discharge was unknown. Finally, owing to a lack of continuous monitoring of blood tests in this study, admission SIRI was evaluated at one-time point, and fluctuation of SIRI was not considered.

## Conclusion

SIRI was found to be a reliable and independent prognostic factor for patients with ACS undergoing PCI.

## Supplementary Material

Supplemental Material

## Data Availability

Data of this study are available from the corresponding author upon request.
