# Supplementary material for: Prognostic value of systemic inflammatory response index in patients with acute coronary syndrome undergoing percutaneous coronary intervention
Source: Ann Med. 2022 Jun 13;54(1):1667–77. doi: 10.1080/07853890.2022.2083671 (PMC9225721; doi:10.1080/07853890.2022.2083671)
Supplement: Supplemental Material [file IANN_A_2083671_SM1433.docx]

Supplemental Table 1: Comparison of C-index among different indicators for MACE

| Variable | C-index | 95% CI |
| --- | --- | --- |
| Monocyte | 0.581 | 0.557 to 0.604 |
| Neutrophil | 0.612 | 0.589 to 0.635 |
| Lymphocyte | 0.529 | 0.505 to 0.553 |
| SIRI | 0.624 | 0.601 to 0.647 |
